
JOURNAL INFORMATION
==============================
NLM Title Abbreviation: ScientificWorldJournal
Journal ID: ScientificWorldJournal
NLM Journal ID: 101131163
Journal ID: TSWJ

The Scientific World Journal

ISSN: 2356-6140
EISSN: 1537-744X
Publisher: Wiley

ARTICLE INFORMATION
==============================
PMCID: PMC5901303
PMID: 17334601
DOI: 10.1100/tsw.2007.16
Article ID: 453181
Article version: 1
Subjects: Clinical Images

Multiple Cysts in the Kidney and Lungs in a Patient with Polycystic Kidney Disease and Lymphangioleiomyomatosis

Lam Anna P.
Corbridge Thomas C. *
Division of Pulmonary and Critical Care Medicine, Feinberg School of Medicine, Northwestern University, Chicago, IL, USA
*Thomas C. Corbridge: tcc734@northwestern.eduAcademic Editor: Daniel Batlle

Publication date: 2007
Electronic publication date: 2007 Jan 26
Volume: 7
First page: 80
Last page: 82
Copyright: Copyright © 2007 Anna P. Lam and Thomas C. Corbridge.
Copyright year: 2007
License: This is an open access article distributed under the Creative Commons Attribution License, which permits unrestricted use, distribution, and reproduction in any medium, provided the original work is properly cited.
License URL: https://creativecommons.org/licenses/by/3.0/

Keywords: lymphangioleiomyomatosis, tuberous sclerosis complex, polycystic kidney disease

==============================
Clinical Image
*TheScientificWorldJOURNAL* (2007) 7, 80–82
ISSN 1537-744X; DOI 10.1100/tsw.2007.16

TheScientificWorldJOURNAL
www.thescientificworld.com

### Multiple Cysts in the Kidney and Lungs in a Patient with Polycystic Kidney Disease and Lymphangioleiomyomatosis

Anna P. Lam, and Thomas C. Corbridge
*Division of Pulmonary and Critical Care Medicine, Feinberg School of Medicine, Northwestern University, Chicago, IL*

**E-mail:** tcc734@northwestern.edu

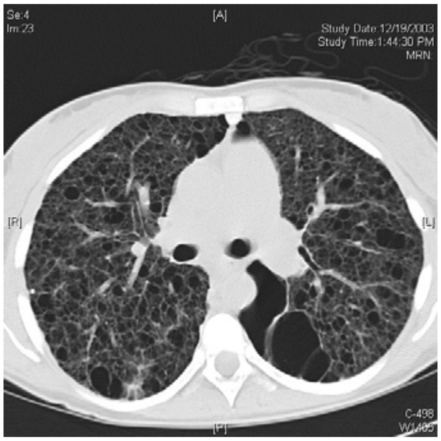

**FIGURE 1.** CT chest demonstrates innumberable thin-walled cysts diffusely distributed throughout both lungs.

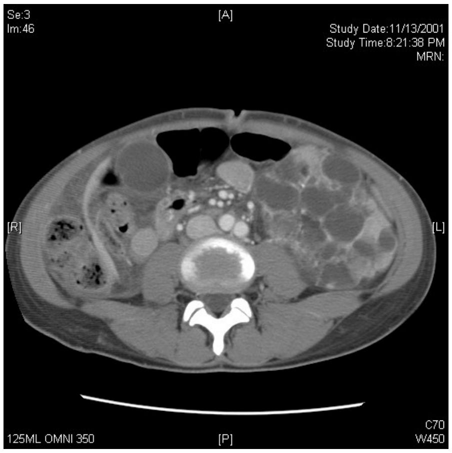

**FIGURE 2.** CT kidney shows a massive left kidney measuring 17.7 cm in cephalocaudal extent with innumerable cysts, interposed by angiomyolipomas.

*Corresponding author.
©2007 with author.
Published by TheScientificWorld; www.thescientificworld.com

**KEYWORDS:** lymphangioleiomyomatosis, tuberous sclerosis complex, polycystic kidney disease

*Received November 15, 2006; Accepted December 12, 2006; Published January 26, 2007*

The following images are from a 32-year-old woman with cystic lung disease, chylothorax, and cystic kidney disease leading to bilateral nephrectomies. CT imaging reveals extensive thin-walled cysts throughout the entire lung parenchyma (Figure 1), while the left kidney is similarly riddled with cysts and angiomyolipoma (Figure 2). Lung biopsy was consistent with lymphangioleiomyomatosis, and renal pathology revealed polycystic kidney with angiomyolipomas consisting predominantly of smooth muscle with areas of fat and vasculature.

Lymphangioleiomyomatosis (LAM), a disease of predominantly women, is characterized in the lung by cysts, pneumothoraces, and chylous pleural effusions. Lymphangioleiomyomatosis (LAM) is characterized by abnormal smooth muscle cell proliferation, leading to cystic destruction of the lung and eventual respiratory failure. Other organ system manifestations include angiomyolipomas in the kidneys, which can also occur in the tuberous sclerosis complex (TSC). TSC, an autosomal dominant neurocutaneous disorder, arises from mutation in the *TSC1* (hamartin) or *TSC2* (tuberin) gene, the latter of which is commonly seen in patients with LAM[1]. One-third of patients with TSC have evidence of pulmonary cysts on imaging of the chest, despite lacking respiratory symptoms[2].

TSC is frequently associated with renal cysts. *PKD1*, which is one of the genes responsible for autosomal-dominant polycystic kidney disease (PKD), lies adjacent to *TSC2*, and deletions in both genes lead to development of the recently described *TSC2/PKD1* contiguous gene syndrome[3,4]. While there have been no studies in the literature thus far documenting a direct association between LAM and PKD, these two diseases and TSC may all be linked by the close proximity of the *TSC2* and *PKD1* genes.

### REFERENCES

1. Glassberg MK. 92004) Lymphangioleiomyomatosis. *Clin. Chest Med*. **25,** 573-82, vii.
2. Pacheco-Rodriguez G., Kristof, A.S., Stevens, L.A., Zhang, Y., Crooks, D., Moss, J., Giles, F., (2002) Filley Lecture. Genetics and gene expression in lymphangioleiomyomatosis. *Chest*, **121**, 56S-60S.
3. Sampson, J.R., Maheshwar, M.M., Aspinwall, R., et al. (1997) Renal cystic disease in tuberous sclerosis: role of the polycystic kidney disease 1 gene. *Am. J. Hum. Genet.* **61,** 843-851.
4. Martignoni, G., Bonetti, F., Pea, M., Tardanico, R., Brunelli, M., Eble, J.N. (2002) Renal disease in adults with TSC2/PKD1 contiguous gene syndrome. *Am. J. Surg. Pathol.*, **26,** 198-205.

**This article should be cited as follows:**

Lam, A.P. and Corbridge, T.C. (2007) Multiple cysts in the kidney and lungs in a patient with polycystic kidney disease and lymphangioleiomyomatosis. *TheScientificWorldJOURNAL* **7**, 80–82. DOI 10.1100/tsw.2007.16.

### APPENDIX

### Editor Comment

Because of the unique importance of these images, which are amenable to different interpretation, we are including the comments of the reviewers.

Daniel Batlle, M.D.

### Reviewer's commentary:

### Ref. 1

While the diagnosis of Tuberous Sclerosis (TSC) is suspected in association with Polycystic Kidney Disease (PKD), the main diagnosis in this patient, the lack of other skin or CNS manifestations to support a diagnosis of TSC opens the possibility that the patient indeed has isolated Lymphangioleiomatosis (LAM) as proposed by the authors. It is important to be aware of the LAM/TSC complex.

Vicente Torres, M.D.
The Mayo Clinic
Rochester, MN.

### Ref. 2

The authors describe interesting images of the lungs and kidneys with cystic formation from a patient who has either Lymphangioleiomatosis (LAM), Tuberous Sclerosis (TSC) or both. The authors favor the diagnosis of isolated LAM with associated angiomyolipomas and polycystic kidney disease (PKD) (three diseases). We favor the possibility that this is a case of LAM/TSC, because PKD is not seen in patients with isolated LAM. By contrast, PKD has been described often with TSC (1,2). Angiomyolipomas are seen in both isolated LAM, and LAM/TSC.

It is important to recognize that some authors consider LAM a partial form of TSC (*form fruste)*, since there is overlap in between these two entities (1,2).

1. Avila, N.A., Dwyer, A.J., Rabel, A., and Moss, J. (2006) Sporadic Lymphangioleiomyomatosis and Tuberous Sclerosis Complex with Lymphangioleiomyomatosis: Comparison of CT Features. *Radiology*, 244-277.
2. Woerner, A.C., Au, K.-S., Williams, A.T., Harris, P.C. Northrup, H. (2006) Tuberous sclerosis complex and polycystic kidney disease together: An exception to the contiguous gene syndrome. *Genetics in Medicine.* **8(3)**, 197-198.

Carla Quijano, M.D.
Saint Francis Hospital
Evanston, IL.

Andres Serrano, MD
Northwestern Memorial Hospital
Chicago, IL.
